# Circulating nucleic acids in the plasma and serum as potential biomarkers in neurological disorders

**DOI:** 10.1590/1414-431X20209881

**Published:** 2020-08-17

**Authors:** D.C.F. Bruno, A. Donatti, M. Martin, V.S. Almeida, J.C. Geraldis, F.S. Oliveira, D.B. Dogini, I. Lopes-Cendes

**Affiliations:** 1 Departamento de Genética Médica e Medicina Genômica, Faculdade de Ciências Médicas, Universidade Estadual de Campinas, Campinas, SP, Brasil; 2 Instituto Brasileiro de Neurociência e Neurotecnologia, Campinas, SP, Brasil

**Keywords:** Circulating nucleic acids, Neurological disorders, Cell-free DNA, Cell-free RNA, Biomarker, CNAs

## Abstract

Neurological diseases are responsible for approximately 6.8 million deaths every year. They affect up to 1 billion people worldwide and cause significant disability and reduced quality of life. In most neurological disorders, the diagnosis can be challenging; it frequently requires long-term investigation. Thus, the discovery of better diagnostic methods to help in the accurate and fast diagnosis of neurological disorders is crucial. Circulating nucleic acids (CNAs) are defined as any type of DNA or RNA that is present in body biofluids. They can be found within extracellular vesicles or as cell-free DNA and RNA. Currently, CNAs are being explored as potential biomarkers for diseases because they can be obtained using non-invasive methods and may reflect unique characteristics of the biological processes involved in several diseases. CNAs can be especially useful as biomarkers for conditions that involve organs or structures that are difficult to assess, such as the central nervous system. This review presents a critical assessment of the most current literature about the use of plasma and serum CNAs as biomarkers for several aspects of neurological disorders: defining a diagnosis, establishing a prognosis, and monitoring the disease progression and response to therapy. We explored the biological origin, types, and general mechanisms involved in the generation of CNAs in physiological and pathological processes, with specific attention to neurological disorders. In addition, we present some of the future applications of CNAs as non-invasive biomarkers for these diseases.

## Introduction

According to the World Health Organization (WHO), neurological diseases are one of the greatest threats to public health: they affect up to 1 billion people worldwide and cause around 6.8 million deaths every year (1). The diagnosis of most neurological disorders is often challenging because of the generally nonspecific clinical presentation and/or the lack of accurate biomarkers, especially in multifactorial diseases (2). Some methods used to investigate neurological disorders are based on lumbar puncture and, when possible, histological findings from tissue biopsies. However, these methods are considered invasive, painful, and potentially dangerous, which makes diagnosis and investigation difficult in some cases (3). In contrast, the collection of peripheral blood can be considered a non-invasive procedure when compared to such methods and can assist in the investigation (3,4). Moreover, the disability caused by most of these neurological disorders is typically devastating. Therefore, a rapid and accurate diagnosis can often save or significantly improve the lives of many patients (2). Faced with this problem, several researchers have searched for better diagnostic methods, as well as new approaches for the diagnosis, establishing a prognosis, and monitoring therapeutic response in different neurological conditions. These endeavors have led to recent reports of the potential use of circulating nucleic acids (CNAs) as non-invasive biomarkers in neurology (5).

**Figure 1:**
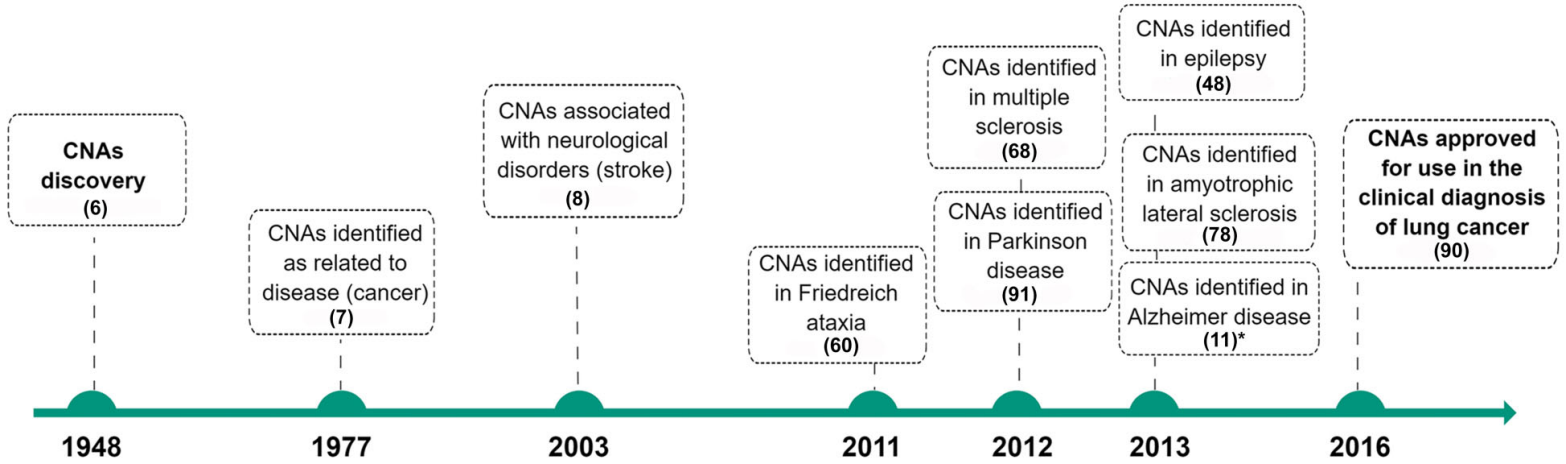
Important milestones for the identification of circulating nucleic acids (CNAs), including their discovery regarding neurological disorders. The timeline shows the main CNA discoveries, including the first report in 1948 (6), the first association of CNAs to disease (7), and the increasing interest seen in the last decade in neurological disorders (8,48,60,68,78,91)(11* Kumar et al., doi: 10.1371/journal.pone.0069807, from Supplementary Table S1), and even the United States Food and Drug Administration has approved the use of CNAs as biomarkers for clinical diagnosis in lung cancer (90). The figures were generated using the software Mind the Graph^®^ (https://mindthegraph.com).

CNAs were first discovered in the 1940s (6) and, in 1977, Leon et al. (7) reported high levels of CNAs in the serum of patients with pancreatic cancer. More than 60 years after their initial discovery, CNAs were first described in neurological disorders such as stroke (8). However, it was only in the past decade that the interest in studying plasma and/or serum CNAs as biomarkers of neurological disorders has increased (Figure 1). Although CNAs can be recovered from other biofluids such as urine, amniotic fluid, saliva, cerebrospinal fluid, milk, etc. (9), we will focus on the current literature about the presence and study of CNAs in the plasma and serum. Thus, this review summarizes the existing knowledge about the use of plasma and serum CNAs as a biomarker to diagnose, establish a prognosis, and monitor the progression and response to therapy in neurological disorders.

## CNAs in the plasma and serum: characterization and potential applications

CNAs refer to any DNA (genomic, mitochondrial, and even from microorganisms) or RNA (all RNAs classes, which will be discussed in more detail later) found in biofluids; they may originate from different cell types (9). The use of CNAs has a potentially important role for both clinical and research purposes, mainly because they can reflect specific characteristics of the biological processes underlying disease; thus, they can act as biomarkers (3,5,9). Biomarkers are defined as measurable indicators of biological processes, either normal or pathological. They may be used for different purposes in relation to disease, including diagnosis, prognosis, therapeutic monitoring, detection of disease recurrence, and susceptibility prediction (10). Biomarkers can be used alone or in combination with other biomarkers and clinical features. Thus, CNAs can be used as biomarkers in the context of predictive, preventive, and personalized medicine in a variety of conditions, including neurological disorders (3,5,9).

Blood (plasma and serum) has been one of the most widely used biofluids for both clinical and research use, mainly due to its abundance and easy access. Both plasma and serum are considered reliable sources. While the former is isolated after centrifugation of anticoagulated blood, the latter represents the supernatant that emerges after blood clotting (11). Although blood is a reliable CNA source, there are several parameters during blood collection and processing that need to be controlled to assure quality and accuracy in the isolation and quantification of CNAs (12). Plasma and serum have the same biomolecular composition, except for fibrinogens and coagulation factors that are absent in serum (13). Nevertheless, studies suggest that serum has a higher CNAs concentration compared to plasma, especially for cell-free DNA (cfDNA), a phenomenon that is probably due to contamination of genomic DNA after cell lysis during the coagulation process. Therefore, plasma appears to be a more reliable source of CNAs, although the quality of the obtained CNAs is highly influenced by the anticoagulant used and inadequate blood processing (11).

The blood-brain barrier (BBB) is a semipermeable membranous barrier located at the interface between the blood and brain tissue. It is responsible for maintaining the central nervous system (CNS) homeostasis, protecting the CNS against toxic insults and pathogens, providing nutrients to the brain, and regulating the peripheral communication with the CNS (14). Under normal physiological conditions, small CNA fragments can cross the BBB and reach the circulating plasma and serum (3,5). However, pathological conditions may cause BBB disruption, an event that increases its permeability and allows the open flow of molecules, cells, and CNAs between the CNS and the peripheral circulation (5,8,14). Moreover, CNAs can be transported through the BBB in a more controlled manner, such as in cell-to-cell spread within extracellular vesicles (EVs) (15). BBB disruption plays an important role in inflammation and cellular damage in many neurological disorders (3,8,14). The identification and quantification of CNAs can reflect pathological processes that occur in the CNS. Thus, by using a non-invasive procedure, blood-derived CNAs can be used as biomarkers for different neurological disorders.

**Figure 2:**
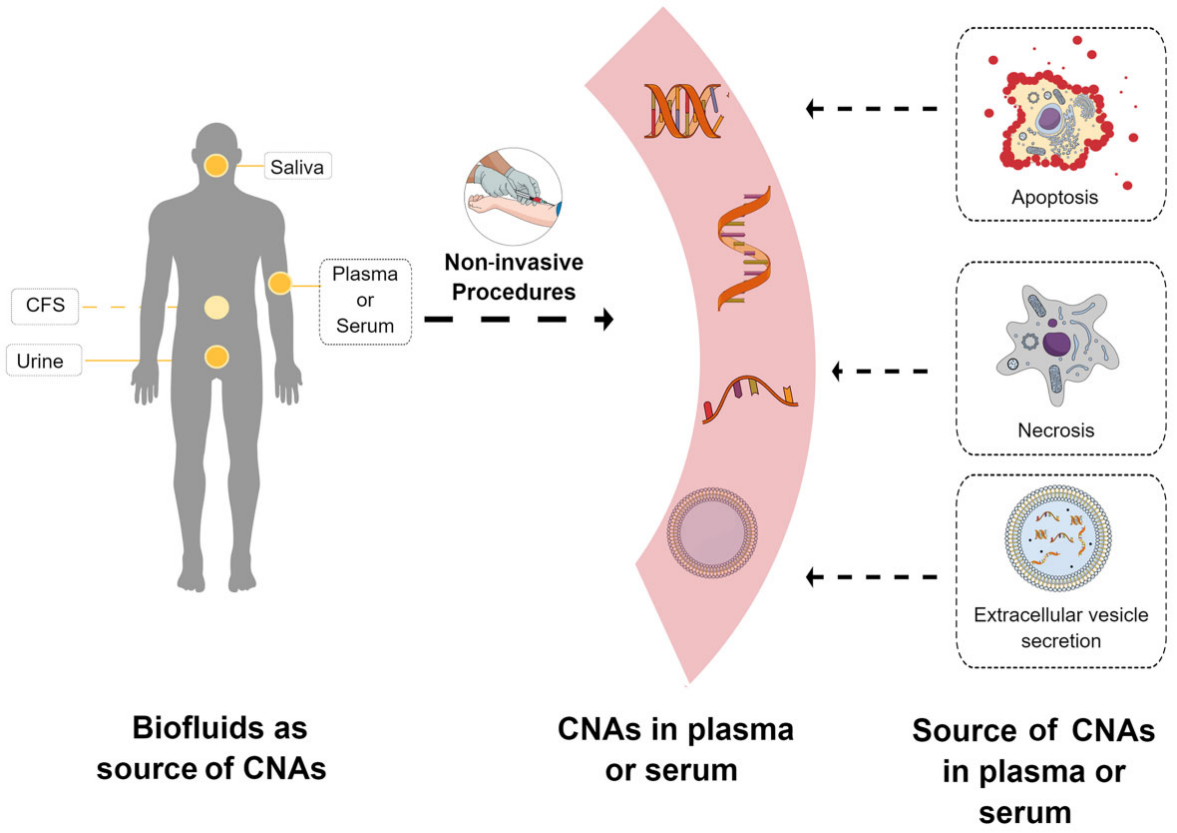
A schematic representation of the different sources of circulating nucleic acids (CNAs) and their detection in biofluids. CNAs (DNA and RNA molecules) can be released into circulation due to various cellular mechanisms, including apoptosis, necrosis, or extracellular vesicles. The release of CNAs can be detected in different biofluids such as saliva, cerebrospinal fluid (CSF), urine, plasma, and serum. This detection requires only non-invasive procedures. The figure was generated using the software Mind the Graph^®^ (https://mindthegraph.com).

The source of CNAs isolated in plasma or serum samples is still a highly debated topic (5,9). Some studies have proposed that CNAs are released into different biofluids by passive mechanisms, such as necrosis and apoptosis, and/or by the active release of nucleic acids from cells (5) (Figure 2). The passive release of CNAs is supported by the DNA fragmentation pattern, which is similar to the pattern of degraded DNA in apoptotic cells (9,16). Thus, it is believed that CNAs from apoptotic cells are exposed to endonucleases and ribonucleases, and this exposure leads to their fragmentation and the consequent disposal associated with nucleosomes or within apoptotic bodies (4,5). Moreover, CNAs can also be released passively during necrosis, a pathological process that results in the random cleavage of DNA and its release into the extracellular medium as larger fragments (5,16).

Some studies have proposed that cells can release CNAs that are actively bound to EVs and/or associated with macromolecular complexes, such as virtosomes (newly synthesized nucleic acids combined with lipoproteins) and ribonucleoproteins (e.g., Argonaute2 complex) (4,17). These structures, along with apoptotic bodies and nucleosomes, protect CNAs from digestion by nucleases, making them stable in biofluids. The amount of CNAs found in different biological states can vary remarkably, depending on the type of biofluid analyzed (9,12). However, in healthy individuals, most of the dying cells are eliminated in phagocytic bodies; thus, only a small amount of CNAs are released (16).

## Extracellular vesicles

CNAs present inside vesicles isolated from biofluids, especially plasma and serum, are protected against enzymatic degradation and can be carried over long distances by vesicular transport (18). These vesicles are referred to as EVs; they are small lipid complexes released from almost all cell types in response to cellular activation (e.g., during cell stress) and/or apoptosis (15). For many years, scientists thought that EVs were predominantly a type of cellular waste; however, more recent studies have shown that they have many other functions, mainly related to cellular homeostasis and maintenance of the cellular environment (15). Furthermore, EVs participate in intercellular communication, transmit protective properties, transfer cellular receptors and genetic cargo, modify cell fate, plasticity, and modulate the immune response (15,18). In addition, they also seem to be involved in tissue regeneration after cell damage. This finding suggests that EVs may also be used for treatment purposes (15).

EVs can be classified into three main types: apoptotic bodies, microvesicles, and exosomes (15). Each type has a distinct origin and size and may be formed by different molecules and mechanisms. Among these, the apoptotic bodies are the largest, around 500–2,000 nm (19); they are irregular in shape and are formed during the late stages of apoptosis (15,19). These apoptotic bodies have a permeable membrane, contain phosphatidylserine on the surface, and carry components of the nucleus and organelles, as well as membrane contents (15). In addition, these apoptotic bodies may induce an anti-inflammatory and/or tolerogenic response to the extracellular environment and thus promote regulation of the immune system (15,19).

Microvesicles, also known as shedding vesicles or ectosomes, are approximately 50–1,000 nm in size. They are formed from cells in the resting state or after suffering stress, such as hypoxia and oxidative stress (15,19). Microvesicles are released by budding out of the membrane surface, followed by a fission event. These microvesicles have specific interactions with target cells and can transfer genetic information from cell to cell (15). In addition, microvesicles can influence the behavior of target cells by direct stimulation, receptor transfer, and protein-protein interactions (15,19).

Exosomes were first described in the 1980s when researchers found a transferrin depletion during the maturation of reticulocytes into erythrocytes (20). However, only in the last decade have exosomes gained the attention of the scientific community (20), namely as key elements in the propagation of different cellular components, such as proteins, lipids, and CNAs. Exosomes are 30–100 nm in size (19) and represent the best characterized EVs (15). They are found in almost all body fluids, specifically inside large multivesicular endosomes (18,21). These vesicles are mainly derived from immune system cells, such as dendritic cells, B cells, and mast cells (15,20). Exosomes are formed through the internalization of the endosome membrane, an action that generates an intraluminal vesicle inside the endosome (18,19,21), which can be released into the extracellular environment or sent to degradation in the lysosome (20,21). When these vesicles are liberated into the extracellular environment, they may carry their contents to other cells and influence several molecular pathways in normal and disease states (20,21). Indeed, exosomes have been implicated in several diseases (18–21).

EVs can transport different molecules from cell to cell (15,22); therefore, EVs can be used in gene therapy and as potential biomarkers of diseases (15,18). EVs can also transport different classes of RNAs (22), and there are currently 27,646 messenger RNA (mRNA) and 10,520 microRNA entries associated with EVs in Vesiclepedia (results obtained July 15, 2020; http://microvesicles.org/index.html) (23). The RNA content of plasma exosomes has recently been well characterized (24) and shown to be very diverse: 40.4% mature microRNAs, 40% Piwi-interacting RNAs, 2.4% long non-coding RNAs (lncRNAs), and 2.1% mRNAs (24) (the details and the different classes of cell-free RNAs (cfRNAs) will be covered in more detail below). These findings indicate their role in gene regulation due to the high content of non-coding RNAs.

By contrast, there are only a few studies of DNA-associated EVs (22). It is believed that specific types of EVs can pack and carry different parts of the genome (22). However, recent evidence indicates that DNA is not carried within EVs; instead, it is connected to their outer surface (22). Therefore, the possible main sources of DNA associated with EVs might be vesicles released by apoptotic cells, cfDNA found in apoptotic bodies, or even cfDNA bound to the surface of EVs (22). Furthermore, some studies demonstrated the presence of different EVs-associated DNA molecules (mitochondrial DNA [mtDNA], single-strand DNA [ssDNA], and double-strand DNA [dsDNA]) in the oncology field (22,25). Thakur et al. (25) found that exosomes can carry the entire genome of tumor cells; this feature can help in the identification of specific mutations from the parental tumor cells. Further, they argued that DNA from tumor-derived exosomes might represent potential biomarkers in the early detection of cancer and metastasis, and as biomarkers for therapeutic monitoring. Moreover, Kawamura et al. (22) reported mtDNA found exclusively on the surface of EVs that were related to glioblastoma and astrocytes.

In the CNS, EVs can be released by different types of cells, including hippocampal neurons, astrocytes, glial cells, and oligodendrocytes (15). These CNS-derived EVs seem to play a role in different communication processes in the CNS; they impact synaptic communications, inflammatory and neurotransmitter signals, cargo transfer among cells and organelles, metabolic activity, and myelin synthesis (15). Furthermore, there is evidence that specific DNA-related EVs are released by distinct cell types in the CNS and have a predetermined target. For example, EVs released by oligodendrocytes are usually endocytosed by neurons, while neuronal EVs might affect the same neuron or an afferent neuron (15). The molecular content of the EVs released by specific cells or organs may help to explain their origin and may be specific in distinct normal or pathological conditions (15). Given that EVs can pass through the BBB, EVs released by CNS cells can be isolated in the peripheral blood. This phenomenon allows for the use of EV contents as potential biomarkers in neurological disorders (8,15).

## Circulating cfDNA

cfDNA may carry disease-specific markers (26); therefore, identification and quantification of cfDNA have been used in clinical practice, mainly in prenatal diagnostics and as biomarkers for different types of cancers (9). In healthy individuals, the main source of cfDNA is believed to be predominately apoptotic progenitor hematopoietic cells, with only a small contribution of cells from other tissues (9,26). Under normal physiological conditions, the concentration of cfDNA in the plasma and serum is very low (10-50 ng/mL) because most non-living cells are efficiently removed from the circulation by phagocytes (9,16). In contrast, cfDNA from different origins can increase under abnormal conditions (9,16,26).

cfDNA may originate from various sources, including nuclear, mitochondrial, or microbial genomes. However, the predominant type that is currently studied is from the nuclear genome, with an average fragment size of 160-180 base pairs (bp) (16,27). The molecular weight and size distribution of cfDNA may indicate its source. Specifically, apoptosis produces fragments around 180 bp, whereas necrosis results in larger fragments (16). Some studies have shown that cfDNA from nucleosomes produces approximately 147 bp fragments, while chromatosomes (nucleosome + histone ligand) produce around 167 bp fragments (26). In addition, cfDNA may circulate as nucleosomes or chromatosomes rather than as an isolated DNA fragment (16,26). Nucleosomes play an important role in DNA fragmentation during apoptosis and may indicate the cfDNA’s origin (24,26). An increased concentration of circulating nucleosomes (cf-nucleosomes) has been associated with biological processes that occur in accelerated cell death, including in degenerative and autoimmune disorders, ischemia, and trauma (28). However, the use of cf-nucleosomes as biomarkers is still limited - mainly restricted to the detection of early-stage cancer (28).

In cancer, high levels of cf-nucleosomes have already been associated with tumor burden and disease progression. Also, aberrant DNA methylation and histone modifications are characteristic of cancers, and these can be present in cf-nucleosomes. These characteristics make cf-nucleosomes potent biomarkers for early detection and monitoring of the disease (28). Therefore, we believe that, as in cancer, modifications of cf-nucleosomes can be useful as biomarkers in neurological disorders, mainly because cf-nucleosomes can give information about the cellular origin and/or specific marks of cellular damage (26).

Recent studies by Burnham et al. (27) argue that cell-free mtDNA (cf-mtDNA) is more abundant in the plasma, specifically 56-fold higher compared to the nuclear genome. This phenomenon is most likely due to the number of mtDNA copies. However, cf-mtDNA is more fragmented than cfDNA that originated from the nuclear genome (27). Although cf-mtDNA has not been explored as a potential biomarker, mtDNA has several advantages compared with nuclear DNA, including: i) mtDNA is small and therefore more amenable to deep sequencing and other types of experimental manipulation; ii) there are thousands of mtDNA copies per cell, and they are abundant in the plasma and serum; iii) the mitochondrial genome is highly polymorphic, a factor that makes it easier to differentiate the distinct mtDNA origins, e.g., donor × recipient (27); and iv) mtDNA may be of special interest in neurological disorders because mitochondrial dysfunction and/or changes in mtDNA have been implicated in many neurological diseases (Supplementary Table S1). Quantification of the levels of cf-mtDNA might have additional significance for the disease mechanism because it is related to cellular oxidative stress and senescence (29).

Furthermore, mtDNA is highly vulnerable to oxidative stress and may reach a mutagenicity rate of 10 to 200 times greater than nuclear DNA; thereby, it can inform researchers about cellular stress and even cellular function based on sequencing information (29,30). Indeed, a significant increase in nuclear and mitochondrial cfDNA occurs in a wide variety of biological processes (9,29,30). The first report of the use of cfDNA as a potential biomarker in a neurological disorder was in stroke (8). Subsequently, additional studies have been published in other neurological conditions (see Supplementary Table S1). Details about specific neurological disorders will be given below. Nevertheless, the use of cfDNA as disease biomarkers has some caveats because the cfDNA concentration might be influenced by many physiological states, such as pregnancy, intense exercise, smoking, trauma, and inflammation (31).

The use of cfDNA as a biomarker goes far beyond its simple quantification in the serum and plasma. In fact, cfDNA may be genetically investigated in the same ways as intracellular DNA. cfDNA sequencing might have additional advantages because there appears to be tissue-defined sequencing specificity of the circulating cfDNA (26), such as chromosomal rearrangements, microsatellite alterations, point mutations, insertions and deletions, multi-nucleotide polymorphisms, loss of heterozygosity, copy number variations, and epigenetic alterations (31). Most efforts in the study of cfDNAs as potential biomarkers have focused on the search for mutations and/or DNA sequence variants. However, the study of epigenetic marks in cfDNA has shown promise in recent years (32).

Epigenetics is influenced by the environment and plays a role in all aspects of neuronal function, from embryogenesis and early development to specific gene expression, as well as gene silencing (33). The main epigenetic mechanisms are DNA methylation, non-coding RNAs, and histone modifications. Epigenetic dysregulation plays a significant role in several disease aspects (33). In addition, the recent expansion of knowledge about epigenetic changes strongly suggests that biomarkers based on epigenetic rather than genetic changes might become more useful biomarkers for the detection and diagnosis of different diseases (32). Disease-specific DNA methylation patterns undergo unique changes in response to treatment, a phenomenon that increases the possibility that DNA methylation-based biomarkers can be used to monitor treatment efficacy, predict the response to treatment, and establish a prognosis after treatment. Although the use of cfDNA as biomarkers for oncology currently represents their most studied application, other medical fields are likely to benefit in the near future, especially as research expands to explore different diseases, as demonstrated by groundbreaking studies of cfDNA methylation in metabolic, neurological, autoimmune, and psychiatric diseases (32). The most studied epigenetic mechanism in cfDNA is DNA methylation, but recent work has shown that changes in the pattern of histone modification in circulating nucleosomes might also serve as biomarkers (28).

**Figure 3:**
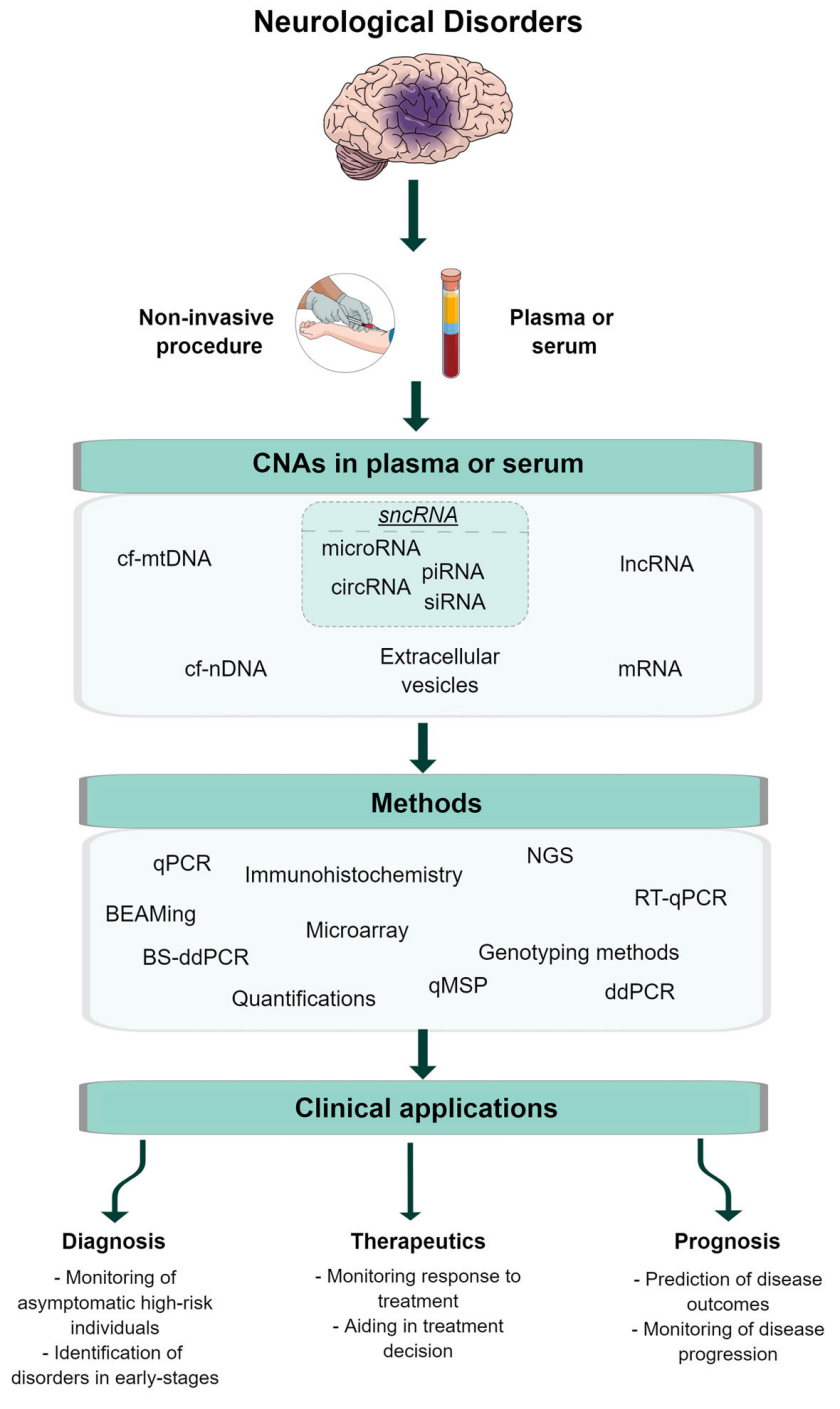
A schematic representation of the potential use of circulating nucleic acids (CNAs) obtained from the serum and plasma in neurological disorders. CNAs in the serum and blood have been described as potential biomarkers for different neurological disorders. In many of these conditions, the central nervous system (CNS) tissue is damaged, a phenomenon that promotes a rupture of the blood-brain barrier (BBB) and subsequent release of CNAs into the peripheral circulation. Consequently, these newly released circulating CNAs will carry the marks of the CNS damage that occurred during the disease process and act as surrogates for the CNS damage. The identification and quantification of the different circulating CNAs could then potentially be used as biomarkers of disease. cf-mtDNA: cell-free mitochondrial DNA; cf-nDNA: cell-free nuclear DNA; lncRNA: long non-coding RNAs; sncRNA: small non-coding RNA; cicRNA: circular RNA; siRNA: small interfering RNA; piRNA: Piwi-interacting RNA; mRNA: messenger RNA; PCR: polymerase chain reaction; qPCR: quantitative polymerase chain reaction; NGS: next-generation sequencing; BS-ddPCR: bisulfite droplet digital polymerase chain reaction; ddPCR: droplet digital polymerase chain reaction; qMSP: quantitative methylation-specific PCR; RT-qPCR: reverse transcriptase quantitative polymerase chain reaction; BEAMing: beads, emulsion, amplification, and magnetics. The figure was generated using the software Mind the Graph^®^ (https://mindthegraph.com). See references: Pérez-Callejo et al., doi: 10.21037/tlcr.2016.10.07; Hovelson et al., doi: 10.18632/oncotarget.21163; Gai and Sun, doi: 10.3390/genes10010032.

Several technical approaches for the analysis of cfDNA have been proposed: quantitative polymerase chain reaction (qPCR); quantitative methylation-specific PCR; droplet digital PCR; bisulfite droplet digital PCR; targeted DNA sequencing; whole-exome sequencing; whole-genome sequencing; whole-genome methylation sequencing; and beads, emulsion, amplification, and magnetics (BEAMing). Furthermore, many other techniques commonly used for the study of cellular DNA may be expanded for the analysis of cfDNA (Figure 3). Nevertheless, despite its great potential, the use of cfDNA as biomarkers is currently limited by the methods available for interrogating cfDNA in biofluids (such as plasma, serum, urine). These methods are limited by low resolution, imperfect precision, and reduced amplitude (5). In particular, searches for single nucleotide variants (SNVs) in cfDNA as biomarkers can be problematic since these fragments are ultra-short. However, although sequencing technologies that can detect new SNVs in cfDNAs are still scarce, increasing the depth of coverage of next generation sequencing methods can help in this problem, as well as new methods that have been implemented, such as HiFRe (high-fidelity short reads method) in MinION sequencing using Nanopore^®^ technology, which proved to be sufficiently accurate to obtain reliable SNV detection (34).

## Circulating cfRNA

The human transcriptome comprises a great variety of RNA molecules, up to 32,000 lncRNAs, more than 20,000 mRNAs, 9,000 small RNAs, and 11,000 transcribed pseudogenes. These diverse species offer a myriad of possibilities for investigating a large set of potential biomarkers for disease (10). In addition to cfDNA, different classes of RNAs may be present in the circulation; they are denoted as cfRNA. These species may show specific expression profiles in different biofluids and distinct biological states and/or disorders (35). However, as opposed to cfDNA, the main source of cfRNAs is attributed to active secretion, communication, and cellular transport rather than cell death (10). cfRNAs can be classified as protein-coding (mRNAs) or non-coding RNAs. Non-coding RNAs are further classified according to size as small non-coding RNAs (sncRNAs), including microRNAs and lncRNAs (35,36). The most recent studies that investigated the application of cfRNAs as biomarkers in plasma and serum have focused on non-coding RNAs, especially microRNAs (35). One possible explanation for the lack of interest in using cf-mRNAs as biomarkers is that although mRNAs play a critical role in many cellular processes and are believed to reflect the status of the intracellular state directly, they are very unstable. Thus, it is difficult to find specific parameters for different pathological states. In addition, cf-mRNA is easily degraded and, therefore, difficult to isolate and quantify (24).

lncRNAs are longer than 200 nucleotides (nt) and resemble mRNAs; however, they do not encode proteins. lncRNAs play a significant role in gene regulation; they control many cellular and molecular mechanisms (reviewed in 36). There are five subclasses: anti-sense, intronic, intergenic, sense overlapping, and bidirectional. In addition to their role in the transcriptional and post-transcriptional control, they act upon gene regulation at the splicing level as well as in chromatin remodeling (24). Being so conspicuous, it is not surprising that lncRNA dysregulation has been associated with the basic mechanisms that underlie different neurological disorders, including Huntington's disease (37) and epilepsy (38). Thus, lncRNAs that are found in the plasma and serum represent potential biomarkers of disease(s), although this possibility has not been fully explored.

sncRNAs are usually shorter than 200 nt. This class includes small nuclear RNAs (snRNA), small nucleolar RNAs (snoRNA), microRNAs, and piRNA (39). MicroRNAs play a role in the post-transcriptional regulation of gene expression in a variety of transcript targets. An interesting aspect of microRNA-mediated gene regulation is its complexity: a single microRNA can regulate many mRNAs, and one mRNA can be regulated by multiple microRNAs (39). Although RNA molecules are relatively unstable and susceptible to degradation by Rnases – a protein superfamily generally abundant in biofluids (10) – studies have shown that microRNAs are particularly stable and resistant to nuclease digestion in the plasma and serum (24,35,36). Thus, microRNAs represent an easily assayed molecule.

As mentioned above, RNA may be present in the plasma and serum as the content of microvesicles; however, part of the RNA in these biofluids is not of a vesicular origin. Indeed, it is estimated that approximately 90% of the microRNAs are transported in a non-vesicular form (40); they are derived from cell death. Moreover, cfRNAs may be released in the circulation associated with protein and lipid complexes. Therefore, the total circulating cfRNA should comprise a mixture of vesicular RNAs, RNAs associated with protein and lipids, and non-associated cfRNA molecules (24). The stability of non-coding RNAs in circulation is thought to be due to the protection offered by microvesicle-associated protein and lipid complexes, which protect against RNase activity (17,24).

MicroRNAs were first detected in the plasma and serum in 2008 (41) and have since been studied as potential biomarkers of disease due to their stability in biofluids. Given that microRNAs can transport information from cell to cell, it is believed that they can reflect modifications in cell content and metabolism, leading to changes in cf-microRNAs released in circulation that could be specific to different diseases (35,42) (for more details see Supplementary Table S1). In addition, small molecules, such as microRNAs, can cross the BBB and enter the circulation; therefore, biological processes that occur in the CNS can be reflected by the pattern and amount of brain-derived microRNAs in peripheral blood (3). MicroRNAs have been associated with the normal function and development of the nervous system as well as neurological disorders (36,42,43). While several studies regarding the use of cf-microRNAs as biomarkers have been published (Supplementary Table S1), there is still much to discover.

As discussed above, biological changes may be reflected in cf-microRNA levels, a phenomenon that makes these species attractive biomarkers for diseases. Furthermore, cf-microRNA levels can be easily quantifiable by RT-qPCR or even by high-throughput techniques, such as microarray or RNA sequencing (RNA-seq) (35,36) (Figure 3). However, the cf-microRNA abundance might be influenced by different biological aspects, such as sex, age, secondary disease, drug treatment, etc. (35), and external factors, as sample collection, processing condition, sample storage, RNA extraction methods, and measurement platforms (35). These internal and/or external factors can significantly affect the results of cf-microRNA quantification and may impact the use of cf-microRNAs as potential biomarkers in clinical practice.

## CNAs in the plasma and serum in neurological disorders

Neurological diseases impact approximately 1 billion people worldwide. They affect people of all ages, races, geographical locations, and socioeconomic status (44). Although neurological disorders have been extensity studied, there is still a wide gap regarding pathogenesis, diagnostic, prognostic, and therapeutic response. Therefore, these diseases represent a significant burden on global health (1,44). Neurological disorders are complex, frequently multifactorial, and thus environmental factors and genetics are involved in the etiology (44). Besides, diagnosis for most neurological disorders is still a challenge because it depends mainly on clinical evaluation and careful patient follow-up. These factors can significantly delay the diagnosis and implementation of the correct treatment. Therefore, the search for disease biomarkers is of paramount importance; however, the anatomical site of the abnormality and the presence of the BBB represents an additional challenge. The most effective methods would non-invasively probe the CNS (3,9). The discovery that CNAs are released into the peripheral circulation and may reflect the biological mechanisms that underlie many neurological disorders has made it possible to investigate their use in the diagnosis, prognosis, and treatment monitoring in different neurological disorders (3,8,14,15,45). Many studies have already demonstrated the use of CNAs in the plasma and serum in neurological disorders, as summarized in Supplementary Table S1 and discussed in more detail below.

### Epilepsy

Epilepsy affects more than 50 million people in the world (46,47). This disease can be progressive and may result in a neurodegenerative and inflammatory reaction, where additional uncontrolled seizures can lead to an increased neuronal loss (48). According to the WHO, 2.4 million people with epilepsy are diagnosed each year, and approximately 30% of these cases are not amenable to treatment with antiepileptic drugs. To date, there is no treatment to prevent or control epileptogenesis (46). An epilepsy diagnosis is based mainly on clinical findings and electrophysiological and imaging tests (49). Although these standard methods are useful, diagnosis remains a considerable challenge for many patients because it requires a certain degree of clinical experience to interpret the findings. Indeed, some studies indicate that misdiagnosis in epilepsy patients occurs in up to 25% of adults (50). The best treatment option for epilepsy is established by the type of epilepsy syndrome (51); thus, a correct and timely diagnosis would allow these patients to receive adequate treatment while avoiding undesirable side effects. Part of the difficulty in achieving accurate diagnosis in epilepsy patients is the complex and multifactorial nature of the condition (which leads to seizures). Therefore, it is currently well recognized that the development of more effective and specific treatments for epilepsy patients strongly depends on biomarker findings, which would allow for a more precise and specific diagnosis and, consequently, lead to an individualized treatment plan (47).

Several studies have demonstrated the potential of using CNAs in the serum and blood as epilepsy biomarkers (see Supplementary Table S1). Studies with cf-microRNA showed that microRNA levels are modulated by the epileptogenic activity (43), and changes in specific cf-microRNAs in the plasma and serum can occur in different types of epilepsy when compared with control samples (52 54) (to consult more studies see Supplementary Table S1). In a recent review, Ma (52) argued that seven cf-microRNAs (miR-134, miR-181a, miR-146a, miR-124, miR-199a, miR-128, and miR-155) have the potential to be used to understand the basic mechanisms in epilepsies and to explore treatment options. Wang et al. (55) suggested that one cf-microRNA (miR-301a-3p) is a potential diagnostic biomarker for refractory epilepsy. Raoof et al. (54) identified at least three cf-microRNAs (miR-27a-3p, miR-328-3p, and miR-654-3p) derived from plasma exosomes as potential diagnostic biomarkers for mesial temporal lobe epilepsy (MTLE). Furthermore, in a case report of sudden unexpected death in epilepsy (SUDEP) due to refractory MTLE, five microRNAs related to drug resistance were investigated from the plasma and hippocampus (autopsy). The study found that miR-301a-3p was positively regulated in the plasma and hippocampus; these data suggest that it might serve as a potential biomarker for SUDEP (53).

There are only two studies published in the last decade that have evaluated circulating cfDNA in epilepsy patients. Liimatainen et al. (48) first showed in 2013 that serum cfDNA levels increased significantly in patients with refractory epilepsy. In 2016, Alapirtti et al. (56) showed that the cfDNA concentration was significantly higher in patients with extratemporal lobe epilepsy compared to healthy individuals. Both studies suggested that increased cfDNA might be associated with the degenerative and inflammatory process that affects the CNS in patients with epilepsy.

### Alzheimer's disease

Alzheimer's disease is a neurodegenerative disorder that affects approximately 46 million people worldwide (40). It is considered the most common cause of dementia in the elderly (57). The diagnosis is based on a combination of clinical criteria and imaging tests; however, there is still a low diagnostic specificity (40). Although imaging tests contribute significantly to an Alzheimer's disease diagnosis, they are conclusive only in advanced stages of the disorder. To date, the most important Alzheimer's disease biomarkers are the tubulin-associated unit (TAU) and the β-amyloid peptides. The levels of both peptides are commonly determined in cerebrospinal fluid (CSF); when combined with neuroimaging exams, these measures can provide excellent diagnostic accuracy. During the early stages of Alzheimer's disease, the levels of these proteins may be inaccurate because they may also be found in other neurodegenerative diseases and exhibit marked variability in their concentrations that do not allow for a specific diagnosis for Alzheimer's disease. In addition, CSF dosing is considered to be an invasive approach (40,58).

Given the limitations of these biochemical assays, current efforts have been made to identify new biomarkers for the early diagnosis of Alzheimer's disease (40). Olsson et al. (58) showed that while it is possible to evaluate minimally invasive β-amyloid and TAU by plasma collection, such proteins do not yield significant diagnostic results that are sufficient to replace the concentration of these proteins in the CSF (58). Some researchers seeking such biomarkers suggest certain cfRNAs found in the plasma and serum might serve as early diagnostic biomarkers of Alzheimer's disease and even as biomarkers for monitoring the progression of the disease (Supplementary Table S1). Pai et al. (57) demonstrated that increased cfDNA levels and methylation of the gene *LHX2* in plasma cfDNA might be useful for early diagnosis of Alzheimer's disease. Furthermore, given that oxidative damage in mtDNA has been well established as one mechanism that underlies the disease (40), it is possible that changes in the levels of cf-mtDNA could be used as a potential biomarker. Mathew et al. (59) pointed out that damage to mtDNA can be detected in the plasma and CSF of patients with Alzheimer's disease. To date, there is no study that shows cf-mtDNA in biofluids such as plasma and/or serum as a potential biomarker, only in CSF. However, such studies remain controversial as to the integrity and amount of cf-mtDNA that can be detected in the CSF (60).

### Parkinson's disease

Parkinson's disease is the second most common neurodegenerative disorder in the world (after Alzheimer's disease). Parkinson's disease is characterized by neuronal loss and the presence of Lewy bodies (abnormal proteins aggregate inside nerve cells) in different brain regions, mainly the substantia nigra. This pathology causes motor dysfunctions and multiple non-motor clinical signs and symptoms (61). Disease onset occurs approximately 10 to 20 years after the initial pathological lesion in the brain, and the diagnosis is predominantly based on clinical evaluation (3). A delay in the diagnosis makes the disease extremely difficult to treat (3), a factor that highlights the importance of identifying new biomarkers for the disease.

Recently, there has been increasing evidence that cf-microRNAs found in biofluids (plasma, serum, and CSF) of Parkinson's disease patients might be useful as biomarkers for early diagnosis, to assess the stage and severity of the disease, and to monitor the disease progression and response to therapy (for more details see Supplementary Table S1). Besides, cf-microRNAs might be useful to establish a differential diagnosis from other diseases that can mimic Parkinson's disease, as reported by Vallelunga et al. (62). In that study, the authors analyzed the serum of healthy individuals, patients with Parkinson's disease, and individuals with multiple system atrophy, a condition that is commonly misdiagnosed as Parkinson's disease. Their results showed that among the 754 analyzed microRNAs, nine cf-microRNAs were differentially expressed when comparing the patient groups with controls. This finding suggests that cf-microRNAs might discriminate Parkinson's disease multisystem atrophy patients (62). Recently, Chen et al. (63) suggested that plasma miR-27a might be a potential biomarker of therapeutic targets in Parkinson's disease.

Most studies that have addressed cfDNA in Parkinson's disease have used CSF, a rather invasive approach (64,65). These reports indicated that cf-mtDNA levels (65) and mutations in the *LRRK2* gene (64) could be used as potential biomarkers for Parkinson's disease. In addition, a study by Chen et al. (45) suggested that increased plasma cfDNA levels would be associated with worse cognitive performance in early-onset Parkinson's disease.

### Multiple sclerosis

Multiple sclerosis is an immune-mediated disorder that leads to demyelination of axons in the CNS. Its prevalence is variable depending on the geographic region; it ranges from 50–400 per 100,000 inhabitants (66). In multiple sclerosis, oligodendrocytes, cells that produce myelin in the CNS, die after an autoimmune response, an action that results in axon demyelination in the brain and spinal cord. Without the myelin, the axons remain exposed and degenerate (67). Multiple sclerosis is a genetically complex disorder in which environmental factors (possibly pathogen-mediated) result in an abnormal immunological response in genetically predisposed individuals (68). A multiple sclerosis diagnosis is primarily based on clinical findings, although magnetic resonance imaging coupled with functional assessment of some specific CNS areas are considered the gold standard for diagnosis. In any case, diagnosis may be especially problematic in the early stages of the disease (69). Recently, several studies have investigated the potential of CNAs as biomarkers in multiple sclerosis (67–69). The first found that there were specific plasma cf-microRNA signatures in multiple sclerosis patients. The data suggested that at least seven cf-microRNAs (upregulated: miR-614, miR-572, miR-648, miR-1826, miR-422a, miR-22; downregulated: miR-1979) might be candidate biomarkers for diagnosis and assist in establishing prognosis in patients with multiple sclerosis (68).

Myelin oligodendrocyte glycoprotein (MOG) is a CNS-specific protein that is only expressed by oligodendrocytes (67). Olsen and collaborators hypothesized that the *MOG* gene might have a specific methylation pattern in oligodendrocytes and might represent a way to assess disease activity in multiple sclerosis (67). They analyzed serum cfDNA and showed a difference in the methylation status in the *MOG* gene in oligodendrocytes from multiple sclerosis patients and in a mouse model of the disease (67). Besides, Dunaeva et al. (69) also argued that cfDNA methylation could serve as a biomarker for recurrent remitting multiple sclerosis. They showed that the methylation level of a subset of the CpG sites within the *LINE-1* gene promoter was hypermethylation in serum cfDNA of patients compared to healthy individuals (69).

### Stroke

Stroke is characterized by blood flow blockage to a part of the brain. This phenomenon results in the loss of neurological functions and, eventually, cell death (70,71). This disorder is considered the second leading cause of death and a major cause of disability in adults, with an important risk factor for the development of dementia (70,71). Furthermore, stroke can be classified into two main types: hemorrhagic and ischemic, with ischemic being the most frequent (∼87%) (70,71). Both types can result in cell death and BBB disruption, a phenomenon that exposes the neuronal content to the circulation (72). An accurate stroke diagnosis can vary considerably (72,73). Stroke misdiagnosis accounts for 40,000-80,000 deaths annually in the USA (74). Establishing the prognosis for stroke patients is as important as an accurate diagnosis; recurrent stroke events are common and tend to increase the odds of mortality and disability and lead to a higher public health cost (74). In this context, it is evident that non-invasive stroke biomarkers are urgently required.

Different biomarker categories have already been studied in stroke (physical, imaging, electrophysiological, histological, genetic, systemic [serum or plasma], and neuronal). However, the heterogeneity of the causes makes their interpretation difficult (75). To our knowledge, there is no simple and accurate blood test that can be used to diagnose and determine the severity of a stroke in patients who present to the hospital emergency unit. During a stroke event, several molecular mechanisms are activated, including cell death, excitotoxicity, and inflammation (70). Given that stroke involves cell death and BBB rupture, CNAs are expected to be released into the plasma and serum soon after the onset (8). The concentration/specific characteristics (e.g., methylation pattern and/or differential expression of microRNAs) might reflect the magnitude of the damage and the initial response to CNS injury and thus serve as a surrogate severity biomarker for the acute stroke event. A pioneering study by Rainer et al. (8) demonstrated that cfDNA concentrations correlate with the severity of the stroke and can be used to predict mortality and morbidity (8). After this initial report, other studies used CNAs as potential biomarkers (Supplementary Table S1). Most of these showed that there is a relationship between cfDNA concentration and stroke (8,72). More recently, cf-microRNAs have emerged as potential stroke biomarkers for stroke (see Supplementary Table S1).

Tiedt et al. (73) suggested that at least three cf-microRNAs (miR-125a-5p, miR-125b-5p, and miR-143-3p) are associated with acute ischemic stroke; these cf-microRNAs might have clinical utility as early diagnostic biomarkers (73). In addition, Vijayan et al. (71) highlighted hundreds of unregulated cf-microRNAs in stroke patients. The authors suggested that these cf-microRNAs and their target genes might be involved in stroke regulation and might serve as potential biomarkers for both therapeutic approaches and stroke diagnosis. They suggested four cf-microRNAs (PC-3p-57664, PC-5p-12969, miR-122-5p, and miR-211-5p) as being of the greatest relevance for the diagnosis of ischemic stroke (71). Despite all the promising results of CNAs as stroke biomarkers, further studies are required to identify the most effective biomarkers, mainly due to the marked heterogeneity of this disorder.

### Amyotrophic lateral sclerosis

Amyotrophic lateral sclerosis is the third most common neurodegenerative disorder worldwide. This incurable disease is always fatal and affects middle-aged individuals (40–60 years old) (76). Amyotrophic lateral sclerosis is characterized by the progressive death of motor neurons in the cortex, brain stem, and spinal cord, with an irreversible deterioration of muscle functions manifested by skeletal muscle weakness and wasting, dysphagia, dysarthria, and respiratory impairment, usually leading to death due to respiratory failure approximately 2–5 years after disease onset (76). Most cases are sporadic, but a small proportion (1–13%) are hereditary (77). The diagnosis is based on clinical examination, electrophysiological findings, medical history, and exclusion of confounding disorders. It is estimated that the diagnostic process in most patients with amyotrophic lateral sclerosis can take more than one year (77). Besides, the most important question patients and family members have after diagnosis is the expected survival of the patient; thus, it is important to identify biomarkers to accelerate the diagnosis and determine the prognosis in patients with amyotrophic lateral sclerosis. Various parameters seen in the disorder have been proposed as biomarkers, including the assessment of oxidative stress, neuroinflammation, metabolic dysfunction, and the underlying neurodegeneration. However, none of these has been incorporated into clinical practice (77). More recently, biomarkers based on CNAs have also been suggested, with cf-microRNAs being the best studied (78–81) (Table 1). De Felice et al. (78) suggested that miR-338-3p, which is found in different biofluids (blood, serum, and CSF), could be used as a biomarker for the diagnosis of patients with amyotrophic lateral sclerosis. Subsequently, Takahashi et al. (79) suggested that two plasma cf-microRNAs, namely miR-4649-5p and miR-4299, might serve as potential biomarkers to establish a prognosis in amyotrophic lateral sclerosis patients. A study by Waller et al. (80) suggested that three cf-microRNAs found in serum – miR-206, 143-3p, and 374b-5p – might serve to monitor disease progression. Another recent study suggested that two cf-microRNAs obtained from plasma-derived EVs, miR-15a-5p and miR-193a-5p, could be related to the diagnosis and progression of disease in patients with amyotrophic lateral sclerosis (81).

There are four main genes – *SOD1*, *FUS*, *TARDBP*, and *C9orf72* – containing variants associated with amyotrophic lateral sclerosis. In addition to these highly penetrating genes, there are at least 100 loci with low penetrance. These findings indicate that a polygenic inheritance is present in most patients with amyotrophic lateral sclerosis (82). In addition, the risk of amyotrophic lateral sclerosis may also be modulated by environmental factors (82). Changes in DNA methylation have been reported in the blood, CSF, and CNS of amyotrophic lateral sclerosis patients (82,83). Furthermore, differential methylation of the *RHBDF* gene in plasma cfDNA of amyotrophic lateral sclerosis patients was found compared with healthy volunteers (83). Although cfDNA from plasma and serum has not been widely studied in amyotrophic lateral sclerosis, it has great potential because this disease causes cell death and damage to the BBB (14).

### Friedreich's ataxia

Friedreich's ataxia is a progressive and degenerative multisystemic disease with an autosomal recessive inheritance. It is considered to be the most common form of hereditary ataxia (84,85). Patients with Friedreich's ataxia commonly have multiple neurological dysfunctions, followed by cardiac disease and diabetes (85). Approximately 95% of Friedreich's ataxia patients are homozygous for an unstable GAA expansion in the *FXN* gene. Heterozygous carriers with no other abnormalities of the *FXN* gene are not considered to be clinically affected, although they have a significant reduction in *FXN* expression (84–186). Friedreich's ataxia is mainly caused by the insufficient production of frataxin, a mitochondrial protein. This protein plays crucial cellular functions: it is essential in iron homeostasis, storage of iron-sulfur clusters, and heme biosynthesis. Therefore, reduced frataxin expression leads to mitochondrial dysfunction (84,86). The breakdown of the BBB, neuronal cell death, and muscle atrophy, all of which occur in patients with Friedreich's ataxia, might be responsible for the release of cfDNA into circulation (84). Although the diagnosis of the disease is relatively straightforward with molecular testing, there are no good biomarkers to determine the prognosis of patients, especially for those who develop systemic complications, cardiac problems, and diabetes.

Therefore, considering that important alterations can be observed in the nuclear and mtDNA of Friedreich's ataxia patients, cfDNA has been investigated in these individuals. Swarup et al. (84) found that plasma cfDNA levels were significantly increased in patients with Friedreich's ataxia and other types of degenerative spinocerebellar ataxia compared to controls. They suggested that cfDNA could serve as a biomarker for the disease. However, they observed that the cfDNA levels did not present a significant correlation with the International Co-operative Ataxia Rating Scale (ICARS) (84). This finding indicates that it is not a good prognostic biomarker. Furthermore, Dantham et al. (87) found that levels of plasma nuclear cfDNA were significantly increased, while cf-mtDNA levels were reduced in patients when compared to controls. Besides, they found that plasma cf-mtDNA had a higher specificity and sensitivity to distinguish between patients and controls. Further, cf-mtDNA changed when patients were submitted to different therapeutic interventions (87). This finding could represent a breakthrough when monitoring the results of clinical trials in these patients.

cf-microRNAs have also been investigated in Friedreich's ataxia. Seco-Cervera et al. (88) sequenced microRNAs from the plasma of Friedreich's ataxia patients and found different cf-microRNA signatures compared to healthy individuals. In addition, a study by Dantham et al. (89) identified the dysregulation of 20 cf-microRNAs in patients. Several of the differentially expressed microRNAs were associated with the pathological mechanisms that underlie the disease, such as CNS injury and early stages of damage as a part of the neuroprotective mechanism (89). Therefore, there is evidence that CNAs in the serum and plasma of Friedrich's ataxia patients might contribute to: i) the development of novel biomarkers; and ii) help better understand the disease pathogenesis.

## Conclusions and Perspectives

The advent of personalized medicine and the possibility of incorporating multiple molecular techniques in clinical practice, such as genomics, transcriptomics, and epigenomics, are likely to improve the diagnosis, prognosis, predictive, and therapeutic monitoring of diseases (31). In the present report, we reviewed the literature on the potential use of CNAs as biomarkers for neurological disorders. Overall, the most significant advantage of incorporating CNAs for clinical use is the ability to use a liquid biopsy – a non-invasive method – to advance the diagnosis, improve the prognosis, and monitor the disease course. Although the last decade saw an increase in publications that investigate the potential role of CNAs as disease biomarkers, the clinical use of these scientific advances has not progressed significantly, except for a limited number primarily in the oncology field (90). In neurological disorders, there is no current example of the clinical applications of CNAs as biomarkers. There are many explanations for this situation, including the fact that extensive validation of scientific findings is required before the recommendation for clinical use. In addition, there is little doubt that, in most cases, a single biomarker will not be enough to achieve the high specificity and sensitivity required for clinical use. Therefore, while CNAs are very promising and important for the study of disease mechanisms, the translation of the current efforts for identifying molecular biomarkers of the disease will require additional efforts from the research and medical communities.

## Supplementary Material

Click here to view [pdf].
